# Diabetes and Reduced Risk for Thoracic Aortic Aneurysms and Dissections: A Nationwide Case-Control Study

**DOI:** 10.1161/JAHA.111.000323

**Published:** 2012-04-24

**Authors:** Siddharth K. Prakash, Claudia Pedroza, Yameen A. Khalil, Dianna M. Milewicz

**Affiliations:** Department of Internal Medicine, University of Texas Health Science Center at Houston, Houston, TX (S.K.P., Y.A.K., D.M.M.); Center for Clinical Research and Evidence-Based Medicine, University of Texas Health Science Center at Houston, Houston, TX (C.P.)

**Keywords:** aorta, aneurysm, diabetes mellitus, risk factors, epidemiology

## Abstract

**Background:**

Vascular diseases are the principal causes of death and disability in people with diabetes. At the same time, studies suggest a protective role of diabetes in the development of abdominal aortic aneurysms. We sought to determine whether diabetes is associated with decreased hospitalization due to thoracic aortic aneurysms and dissections (TAAD).

**Methods and Results:**

We used the 2006 and 2007 Nationwide Inpatient Sample (NIS) to determine TAAD discharge rates. Control subjects were randomly selected to achieve three controls per case. Predictor variables in multilevel logistic regression included age, race, median income, diabetes, and hypertension. We estimated that the average rate of hospital discharge for TAAD among individuals diagnosed with diabetes was 9.7 per 10 000, compared to 15.6 per 10 000 among all discharges. The prevalence of diabetes was substantially lower in TAAD (13%) than in control (22%) records. After adjustment for demographic characteristics, the negative association between diabetes and TAAD remained highly significant in both NIS datasets. Compared to discharges without diabetes, those with chronic complications of diabetes were least likely to be diagnosed with TAAD (OR [odds ratio] 0.17, 95% CI, 0.12–0.23). A significant association remained between uncomplicated diabetes and TAAD. We replicated these findings in an independent group of patients who were hospitalized with acute thoracic aortic dissections.

**Conclusions:**

The principal implication of our findings is that diabetes is independently associated with a decreased rate of hospitalization due to TAAD in proportion to the severity of diabetic complications. Future studies should consider diabetes in predictive models of aneurysm expansion or dissection. **(*J Am Heart Assoc*. 2012;1:jah3-e000323 doi: 10.1161/JAHA.111.000323.)**

## Introduction

Thoracic aortic aneurysms and dissections (TAAD) are among the top 20 causes of death in the United States.^1^ Progressive enlargement of the aorta is usually asymptomatic until a catastrophic event occurs, typically an acute aortic dissection leading to pericardial tamponade, stroke, acute aortic regurgitation, hemothorax, paraplegia, peripheral ischemia, or other complications.^2^ The annual risk of sudden death from an enlarged thoracic aneurysm due to an acute aortic dissection is more than 10%.^3^ Timely surgical repair of aneurysms can prevent death. However, the age of onset, location, and growth rate of aneurysms are highly variable.^4^ Prediction of dissections by current models that primarily consider aortic diameter as a risk factor is not accurate.^5^ A link between other risk factors and aortic dissection has been reported in previous studies. For example, hypertension, substance abuse (cocaine, tobacco, amphetamines), connective tissue disorders (Marfan syndrome and Ehlers-Danlos syndrome), family history of thoracic aortic disease, congenital disorders (bicuspid aortic valve and aortic coarctation), and vascular inflammation (giant cell arteritis and Takayasu arteritis) are presumed risk factors for aortic dissection.^6–8^

Diabetes is an established risk factor for peripheral, coronary, and cerebrovascular disease but was unexpectedly found to be associated with decreased risk for progression and rupture of abdominal aortic aneurysms.^9–12^ The prevalence of diabetes among patients with abdominal aortic aneurysms was consistently found to be lower than comparison groups in diverse clinical settings, leading to the hypothesis that diabetes may have a protective role in the development of abdominal aneurysms. We reasoned that diabetes may also decrease risk for TAAD. Although thoracic and abdominal aortic diseases have distinct clinical and genetic profiles,^13–16^ our hypothesis is supported by observations of decreased atherosclerosis and cardiovascular risk factors that are associated with diabetes in TAAD patients.^17–20^ Appropriate comparisons between a sufficiently large population of TAAD patients and controls with a uniform assessment of diabetes are not feasible using currently available single-center or registry data. Therefore, we used a nationwide sample of hospitalized patients to determine the association between diabetes and TAAD after adjusting for known risk factors.

## Methods

### Data Source

Data are from the Nationwide Inpatient Sample (NIS), collected under the Healthcare Cost and Utilization Project in 2006 and 2007, a product of the Agency for Healthcare Research and Quality (http://www.hcup-us.ahrq.gov/nisoverview.jsp). The NIS is designed to approximate a 20% sample of all nonfederal, short-term general and specialty hospitals in the United States. The sampling strategy selects hospitals within participating states according to defined strata on the basis of ownership, bed size, teaching status, urban/rural location, and region. The 2007 NIS has discharge-level information on primary and secondary diagnoses and procedures and demographics on all discharges from 1000 hospitals in 38 states. Each discharge must be considered independently, as data elements that could directly or indirectly identify individuals are excluded. The NIS contains sampling weights, including strata, primary sampling unit, discharge-level probability weight, and a finite population correction factor, thereby enabling the calculation of national estimates using these data.

### Covariates

NIS data are derived from diagnosis codes (International Classification of Diseases, 9th Revision, Clinical Modification or *ICD*-9-CM, where *ICD*-9 is The International Classification of Diseases, 9th revision) and procedure codes (Current Procedural Terminology or CPT) that are assigned by medical coders who review hospital discharge summaries. Information included in the NIS is contained in a typical discharge abstract. For instance, the *ICD*-9-CM codes 305.1, 989.84, V1582, and 649.0 were used to identify smokers. Other diagnostic codes used in this study are summarized in Table 1. The NIS is the most reliable source of data on US inpatient stays because the data elements are extensively corroborated using multiple methods.^21^

**Table 1.** *ICD*-9-CM* Codes Used to Identify TAAD, Diabetes and Risk Factors

Condition	*ICD*-9-CMCodes
Thoracic aortic aneurysms or thoracic aortic dissections (TAAD)	441.01, 441.1, 441.2

Diabetes mellitus, uncomplicated	250.00–250.33

Diabetes mellitus, complicated	250.40–250.93

Substance abuse	304.20–304.42, 305.60–305.72

Hypertension	401.0–405.99

Tobacco	305.1, 989.84, V1582, 649.0

Marfan syndrome	759.82

Aortic valve disorders†	395.0, 395.2, 395.9, 396.0–396.3, 396.8, 396.9, 397.0, 424.1, 746.3, 746.4

Obstructive sleep apnea	780.51–780.57, 327.20–327.29

Chronic obstructive pulmonary disease	491.0–492.8, 493.2, 494.0, 494.1, 496, 518.1

Ischemic heart disease	412, 414.00–414.07, 414.2–414.9

Chronic kidney disease	582.9, 585.3–585.9, 586

* The International Classification of Diseases, 9th revision, Clinical Modification.

† Includes bicuspid aortic valve

Demographic covariates included age, sex, median income, and race. We treated the median income of the discharge's residence zip code as a categorical variable with four income categories ($1 to $35 999; $36 000 to $44 999; $45 000 to $58 999; >$59 000.) Race was systematically missing from certain hospitals and states (25% of total). Therefore, we ran two versions of our logistic analysis including and excluding race as a covariate. Discharges with a diabetes *ICD*-9-CM code (250.xx) among any diagnosis field were considered to have diabetes. We also identified discharges with diabetic complications (250.4, diabetes with renal manifestations; 250.5, diabetes with ophthalmic manifestations; 250.6, diabetes with neurological manifestations; 250.7, diabetes with circulatory disorders; 250.8, diabetes with other specified manifestation; 250.9, diabetes with unspecified complication); all other discharges with diabetes were considered to have uncomplicated diabetes.

### Case and Control Subject Selection

Discovery cases were selected from among all discharges (alive or deceased) in the 2006 NIS with a diagnosis of thoracic aortic aneurysms or thoracic aortic dissections (*ICD*-9-CM codes 441.01, 441.1, 441.2) in any diagnosis field and age >30 years. Three control subjects were selected for each case by simple random sampling of noncases >30 years. This ratio was chosen to reduce the computational burden of our analyses while maintaining a power of at least 0.90 to reject the null hypothesis when the odds ratio (OR) of diabetes in TAAD cases is ≤0.8. Our estimates were based on recent data demonstrating a similar reduction in the rate of diabetes among patients with abdominal aortic aneurysms.^10^ Replication cases and control subjects were selected in an identical manner from the 2007 NIS. An additional independent replication group consisted of patients >30 years of age who were hospitalized with acute thoracic aortic dissections (UTHSC-H group). Age-matched controls for UTHSC-H patients were selected from patients admitted for chest pain due to coronary artery disease to the Texas Heart Institute at St. Luke's Episcopal Hospital and the Methodist Hospital. Cardiovascular risk factors and clinical data for controls were obtained from the TexGen database at the UTHSC-H Center for Clinical and Translational Sciences as previously described.^22^

### Statistical Analysis

Exploratory data analysis was performed using cross-tabulation of *ICD*-9-CM codes in NIS fields DX1 to DX15, which were ranked by summary statistics (χ^2^ tests). The unit of analysis was the hospital discharge, as we lacked data to identify individuals who had been admitted with TAAD on two or more occasions. Multilevel logistic regression with random effects for hospital variability was used to calculate adjusted OR.^23^ As the authors recommend, we did not use the sampling weights in the logistic analyses but rather included the stratification variables (geographic region, hospital control/ownership, location, teaching status, and bed size) in all models. Clinical risk factors with independent evidence for involvement in the pathogenesis of TAAD (age, sex, diabetes, Marfan syndrome, hypertension, tobacco use, chronic kidney disease, obstructive sleep apnea, chronic obtrusive pulmonary disease, chronic ischemic coronary disease, valvular diseases, and drug use) were also entered into the logistic model. All significance tests were two sided and there was no adjustment for multiple comparisons. Analyses were performed using SAS 9.2 (SAS Institute, Cary, NC). National estimates of discharges with TAAD were generated by applying the provided sampling weights to the sampled discharges in the NIS using SAS survey estimation commands as described by Houchens and Elixhauser.^24^

## Results

### Identification of Case and Control Subjects

We identified 8877 discharges with TAAD (*ICD*-9-CM 441.01, 441.1, or 441.2) from among 8 074 825 discharges in the 2006 NIS database. Of these, 0.6% of TAAD discharges were coded as having the diagnosis of Marfan syndrome and 3.9% were diagnosed with a bicuspid aortic valve, the two most prevalent predisposing conditions for TAAD. The mean age of cases was 70.2±13.9 years, whereas the mean age of 27 069 control subjects was 62.5±17.3 years. Cases were more likely to be hypertensive and tobacco users. A greater percentage of cases compared to control subjects were male and lived in upper median zip codes (Table 2).

**Table 2.** Characteristics of TAAD Cases and Controls

Characteristic	All NIS TAAD	Primary NIS TAAD	NIS Controls	UTHSC-H TAAD	TexGen Controls
*n*	7107	2241	24 148	405	1013

Age (y)	70.1±14.1	66.3±14.1	62.3±17.3	61.8±12.9	62.3±10.9

Race (%)					

White	57.2	50.6	52.0	77.5	79.5

Black	9.2	11.0	10.4	15.3	11.3

Hispanic	4.4	4.9	7.7	5.4	10.5

Missing	24.4	28.9	25.8	0.0	0.0

Male	54.8	56.8	42.7	67.4	67.9

BMI (kg/m^2^)	NA	NA	NA	27.7±6.2	29.4±6.2

Median income (%)					

$1–$38 999	23.6	25.8	28.2	NA	NA

$390 00 0–$47 999	26.3	27.0	25.7	NA	NA

$48 000–$62 999	25.3	24.3	24.5	NA	NA

>$63 000	24.8	22.8	21.5	NA	NA

Bicuspid aortic valve (%)	3.9	3.4	0.02	12.4	0.7

Marfan syndrome (%)	0.6	0.8	0.01	4.7	0.1

Drug abuse (%)	1.8	2.8	3.2	NA	0.1

Hypertension (%)	70.4	71.3	50.3	88.5	69.4

Tobacco (%)	22.3	24.5	17.5	65.4	57.4

COPD (%)	24.3	21.1	14.6	29.9	21.9

Diabetes (%)	13.0	10.7	21.6	15.8	35.9

Ischemic heart disease (%)	36.3	26.7	22.7	37.3	100.0*

NIS, Nationwide Inpatient Sample; UTHSC-H, patients admitted with acute thoracic dissections at the University of Texas Health Science Center at Houston; TexGen, controls without a history of aortic disease from the Center for Clinical and Translational Sciences/TexGen biobank; primary diagnosis of thoracic aortic aneurysms and dissections (TAAD) was based on the first diagnostic field (DX1).

NIS data are from unique discharges in 2006 on the basis of age, sex, race, income, and hospital ID. Data for race represent 75% of discharges reporting race. Data for income reflect 97% of discharges reporting median income of zip code of discharged patient. BMI data were unavailable from NIS and income data were unavailable from UTHSC-H or TexGen.

* TexGen controls were selected from patients who were admitted to the hospital due to ischemic coronary disease.

### Exploratory Analysis

We identified 231 diagnoses that were enriched or depleted in cases compared to controls by cross-tabulation of *ICD*-9-CM codes. For increased specificity, we limited this analysis to 5425 records with a primary diagnosis of TAAD from the 2006 and 2007 NIS datasets. These were grouped into 10 diagnostic categories (with a total of 47 *ICD*-9-CM codes) after exclusion of diagnostic codes for aneurysms or dissections, postoperative complications, malignancies, bone and joint diseases, gastrointestinal diseases, and psychiatric disorders. We found that the prevalence of diabetes showed the most consistent disparity between cases and controls. Eleven different diagnostic codes representing diabetes were uniformly decreased in TAAD cases compared with age-matched controls: 250.00, V58.67, 250.60, 357.2, 790.29, 250.40, 250.80, 250.02, 250.50, 362.01, and 250.70.

### Discharge Rates

To calculate national hospital discharge statistics, we restricted our analysis to individuals who were more than thirty years old in order to exclude patients with congenital causes of TAAD who have distinct clinical profiles. We estimated that in 2006, there were 5700±365 hospital discharges with diabetes and TAAD nationwide in this age group. Dividing this number by the estimated total of 6 000 000±1 40 000 discharges of diabetic individuals in the 2006 NIS database, we estimated that the annual discharge rate for TAAD is 9.5 per 10 000 persons diagnosed with diabetes (95% CI, 8.7–10.3). In the 2006, NIS database the estimated number of all discharges with TAAD was 43 540±2320 and the total number of weighted discharges of individuals above age 30 was 2 7900 000±600 000. Therefore the overall rate of hospital discharge with TAAD was 15.6 per 10 000 (95% CI, 14.5–16.8), which is 40% higher than for diabetic patients.

### Case-Control Analyses

TAAD cases were 40% less likely to have diabetes than control subjects. After adjusting for clinical risk factor differences, diabetes remained significantly and negatively associated with TAAD (OR 0.48, 95% CI, 0.44–0.52). Valvular disease, Marfan syndrome and hypertension were the only predictive factors that were more significant than diabetes in our model. The association between diabetes and TAAD also remained significant in the subgroups of discharges with a primary diagnosis of TAAD (32%, Table 2) or diabetes (3.8%). When our analysis was confined to the remaining cases with diabetes or TAAD as a secondary diagnosis, our results did not significantly change. We also tested for 2-way interactions between diabetes and the other covariates in the model. None of these interactions were significant except for diabetes × sex (*P*=0.013), which indicated that the relationship between TAAD and diabetes was slightly weaker in women than men (OR 0.66 vs. 0.52).

**Figure 1.**
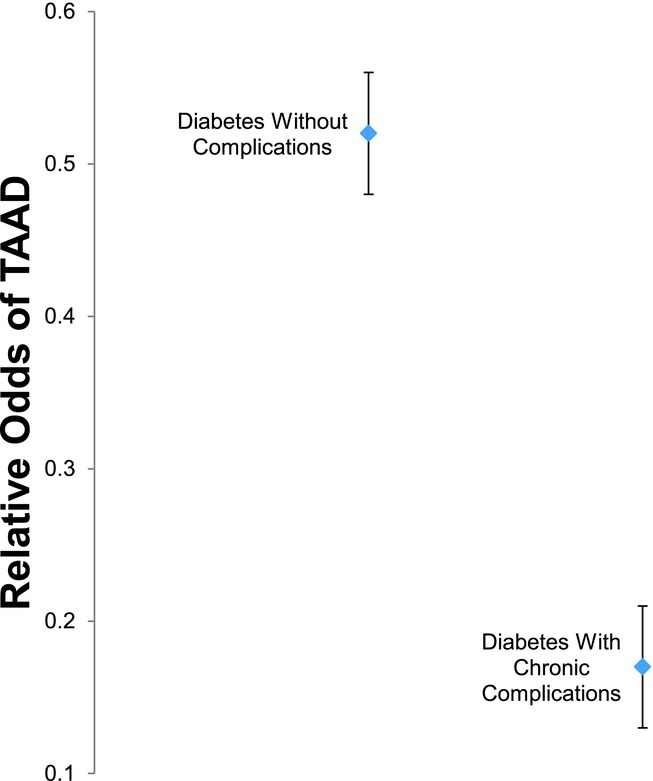
Plot of odds of hospital admission with TAAD as primary diagnosis by diabetic subgroups. *x*-Axis: diabetic subgroups (with or without end-organ complications). *y*-Axis: adjusted odds ratio (OR) of hospital admission in comparison to patients without diabetes.

To minimize the potential for bias related to multiple discharges of the same individuals, we performed an analysis in which we attempted to exclude hospital readmissions. We ascertained that there were at least 7107 unique TAAD discharges in the 2006 NIS (on the basis of unique combinations of age, sex, race, income stratum, and hospital). When limiting analyses to these cases, the relationship between diabetes and TAAD did not appreciably change (adjusted OR 0.47, 95% CI, 0.43–0.51, Table 3). To test the hypothesized relationship between diabetic microvascular disease and TAAD, we performed a subsidiary analysis with diabetes status partitioned into three categories: nondiabetic, diabetes with chronic complications, or uncomplicated diabetes. Compared with discharges without diabetes, those with complications were least likely to have TAAD (OR 0.17, 95% CI, 0.12–0.23). A significant association remained between uncomplicated diabetes and TAAD (OR 0.50, 95% CI, 0.46–0.55; Table 3, Figure 1). In contrast to the strong negative relationship between diabetes and TAAD, we found a significant positive association between diabetes and chronic ischemic heart disease (OR 1.38, 95% CI, 1.25–1.53) in TAAD patients. Because aneurysms and dissections may represent distinct pathological states, we repeated each analysis separately for TAAD cases with thoracic aortic dissections (*ICD*-9-CM code 441.01) and aneurysms without evidence of dissection (*ICD*-9-CM code 441.2). Using the same multivariate models, we found that diabetes remained negatively associated with each subgroup, but the negative relationship between diabetes and TAAD was significantly greater among patients with dissections (OR 0.38, 95% CI, 0.32–0.44) than among patients with aneurysms (OR 0.49, 95% CI, 0.46–0.55; *P*<0.01).

**Table 3.** Odds Ratio of TAAD by Selected Characteristics

Risk Factor	Univariate	Multivariate
All diabetes	0.56 (0.52–0.60)	0.47 (0.43–0.51)

Diabetes without complications	0.60 (0.56–0.65)	0.50 (0.46–0.55)

Diabetes with complications	0.21 (0.15–0.29)	0.17 (0.12–0.24)

Marfan syndrome	40 (12–133)	41.7 (12.1–144.2)

Female gender	0.65 (0.62–0.69)	0.65 (0.61–0.70)

Hypertension	2.35 (2.22–2.50)	2.04 (1.91–2.19)

Tobacco	1.25 (1.16–1.34)	1.32 (1.22–1.43)

Chronic kidney disease	1.28 (1.16–1.40)	1.07 (0.96–1.20)

Obstructive sleep apnea	0.96 (0.81–1.12)	0.89 (0.73–1.07)

Chronic obstructive pulmonary disease	2.04 (1.91–2.19)	1.62 (1.50–1.75)

Ischemic heart disease	1.80 (1.69–1.91)	1.15 (1.07–1.23)

Aortic valve disorders	6.09 (5.64–6.57)	5.45 (5.03–5.91)

Drug abuse	0.56 (0.47–0.68)	1.06 (0.86–1.32)

Race		

Asian	1.48 (1.24–1.76)	1.76 (1.26–2.45)

African American	0.89 (0.81–0.97)	1.05 (0.81–1.37)

Hispanic	0.53 (0.46–0.60)	0.77 (0.58–1.02)

White	1.33 (1.24–1.42)	0.91 (0.71–1.16)

Hospital type		

Small bed size	0.69 (0.59–0.81)	0.65 (0.55–0.75)

Medium bed size	0.91 (0.78–1.06)	0.90 (0.78–1.04)

Government	0.73 (0.53–0.99)	0.79 (0.56–1.12)

Private/nonprofit	0.87 (0.66–1.14)	0.73 (0.52–1.05)

Private/investor-owned	0.85 (0.64–1.13)	0.81 (0.56–1.17)

Rural	0.65 (0.56–0.76)	0.70 (0.57–0.87)

Located in northeast	1.08 (0.87–1.33)	0.95 (0.74–1.21)

Located in midwest	0.98 (0.81–1.19)	0.91 (0.73–1.14)

Located in south	0.76 (0.64–0.92)	0.85 (0.71–1.02)

Teaching	0.62 (0.54–0.71)	0.66 (0.55–0.80)

Data are adjusted odds ratios (95% CI). Data are derived from a multilevel logistic model on the basis of unique hospital admissions in the 2006 Nationwide Inpatient Sample (NIS) database. Significant predictive factors are underlined.

TAAD, Thoracic Aortic Aneurysms and Dissections.

### Replication

We replicated our findings using data from the 2007 NIS, which contains 6825 unique TAAD discharges for an overall discharge rate (15.6 per 10 000 discharges) that is equivalent to the 2006 NIS dataset. In our logistic model with 22 463 control subjects, we again demonstrated an inverse association between diabetes and TAAD (OR 0.47, 95% CI, 0.43–0.51). The relationship was also stronger among subjects with complicated diabetes (OR 0.26, 95% CI, 0.20–0.35) than among subjects with uncomplicated diabetes (OR 0.50, 95% CI, 0.45–0.54). TAAD discharge calculations on the basis of 2007 NIS data are consistent with our initial findings of a 40% decrease in TAAD hospitalizations among diabetic patients (OR 0.63, 95% CI, 0.54–0.73).

We also validated our findings in an independent contemporary dataset of hospitalized patients with acute thoracic aortic dissections (UTHSC-H group). These 405 individuals were matched with 1013 control subjects without any history of aortic disease from the UTHSC-H TexGen biobank. On average UTHSC-H dissection patients are 8 years younger than NIS TAAD patients, with a greater percentage of African Americans and more frequent vascular risk factors. In addition, income and drug abuse variables that are present in NIS were not available in the UTHSC-H dataset. However, after adjustment for all available predictive factors in our multivariate logistic model, diabetes remained a strong independent protective factor against TAAD in UTHSC-H patients (OR 0.38, 95% CI, 0.24–0.52). These findings confirm that diabetes is reproducibly associated with reduced rates of hospitalization due to TAAD in diverse populations with thoracic aortic disease.

## Discussion

These results support the following conclusions. First, hospitalization due to TAAD occurred at a significantly lower rate among individuals with diabetes than among nondiabetic inpatients. Second, diabetes was inversely associated with TAAD independent of age, income, region, hospital type, or other clinical characteristics. Third, the inverse relationship between diabetes and TAAD was present in men and women, in nonwhites and whites, and in cases with and without aortic dissections. Fourth, the inverse relationship between diabetes and TAAD was strongest among discharges with chronic complications of diabetes, suggesting a possible link to hyperglycemia in terms of duration, severity, or susceptibility to vascular injury. Finally, this relationship was quite specific to TAAD.

The strengths of our study included the selection of case and control subjects from a uniform nationally representative database, making information bias unlikely, a rigorous case definition and the selection of a random sample of all control subjects. The principal limitation of this study was our use of hospitalized rather than population-based controls, which may introduce significant bias into our estimates due to enrichment of diabetes among inpatients. We found that this is unlikely, because the overall prevalence of diabetes among unique 2006 NIS discharges (21.6%) was not significantly different than the reported age-adjusted prevalence of diabetes among US adults in contemporary National Health and Nutrition Examination survey data (20.3%).^25,26^ The relationship between diabetes and TAAD also remained constant in an independent cohort of locally recruited inpatients with more prevalent diabetes (35.9%). Our hypothesis is based on cross-sectional associations and requires validation in cohort studies in order to establish a causal relationship between diabetes and TAAD. However, moderate effects of diabetes on clinical endpoints are not likely to be detected in previously published TAAD cohorts due to relatively short-term followup periods and small numbers of patients. This is the largest study of TAAD to date and the only study that is adequately powered to address this question.

The NIS does not contain detailed clinical information on the severity of illness or indications for procedures. This raises concerns about the potential for differential misclassification. We used the same *ICD*-9-CM codes for TAAD as a prior study of nationwide data, and we verified that cases were enriched for procedure codes that are exclusive to TAAD.^8^ Cardiovascular diagnoses such as CHF, hyperlipidemia, ischemic coronary disease, and diabetes were verified to be highly specific when clinical and claims data were compared,^27^ while demographic variables are unlikely to be misclassified in a differential manner with respect to TAAD. Our results may underestimate the impact of diabetes on TAAD because cases may have been more likely to be diagnosed with diabetes than control subjects. This type of misclassification could occur because diabetes may be scrutinized in TAAD patients as a well-known risk factor for vascular disease and would tend to bias our results toward the null hypothesis. Nonetheless, a significant association remained between diabetes and TAAD. Furthermore, we found a positive association between diabetes and other types of vascular disease in the same individuals.

Because no unique personal data were included, we could not identify individuals with multiple hospitalizations. Therefore our results are mainly confined to discharges rather than individuals, and we report discharge rates per population rather than incidence or prevalence per population. When we limited our analysis to a subset of unique discharges, the associations did not substantially change. Although our results with regard to diabetes and TAAD are unprecedented, our findings are consistent with prior studies showing that the rate of abdominal aortic aneurysm progression is reduced in diabetic patients.

Diabetes was recently shown to decrease the progression of aortic disease by direct metabolic effects as well as modulation of inflammation in the aortic wall. In mice with experimentally induced hyperglycemia, the expansion rate of abdominal aortic aneurysms was significantly attenuated.^11^ In clinical studies, diabetes was found to be associated with a reduced prevalence and expansion rate of abdominal aortic aneurysms, and this was correlated with decreased secretion of metalloproteinases by aortic inflammatory cells from diabetic patients.^12,28^ Hyperglycemia is also associated with reduced adventitial neovascularization and decreased infiltration of inflammatory cells into the medial layer of the aorta.^29^ These processes could also inhibit the progression of TAAD by reduction of vascular smooth muscle cell death and extracellular matrix degradation. Alternatively, it is possible that TAAD may protect against the development of diabetes. TAAD is associated with increased circulating concentrations of insulin-like growth factor 1, an endocrine peptide with potent antidiabetic effects.^30,31^ Further experimental evidence will be required to determine the plausibility of these various pathophysiologic explanations for our findings.

We found that diabetes is associated with a 40% to 80% reduction in the rate of hospitalization due to TAAD and is as important as hypertension as a predictor in our multivariate model. Using NIS data, we estimate that TAAD was diagnosed in at least 15 per 100 000 US adults in 2006. This compares favorably with annual estimates of 10.5 to 14.8 cases of TAAD per 100 000 persons in Rochester, Minnesota between 1980 and 1994 and 10.7 to 16.3 cases per 100 000 persons in Sweden between 1987 and 2002.^32,33^ The prevalence of TAAD is increased 10-fold in hospitalized patients compared with the general population, indicating that TAAD is a major cause of morbidity and mortality. The principal implication of our findings is that diabetes is negatively associated with hospitalization due to TAAD. Patients with diabetic complications related to the severity or persistence of diabetes have the lowest rates of TAAD. Future research should seek to determine whether there is a direct vascular protective effect of hyperglycemia on the aortic wall and to disentangle the molecular pathways that mediate this effect. Another implication is that future studies of TAAD treatment should consider diabetes in predictive models of aneurysm expansion or dissection.
